# Data on the expression of leptin and leptin receptor in the dorsal root ganglion and spinal cord after preganglionic cervical root avulsion

**DOI:** 10.1016/j.dib.2017.10.005

**Published:** 2017-10-07

**Authors:** Kai-Ting Chang, Yi-Lo Lin, Chi-Te Lin, May-Jywan Tsai, Wen-Cheng Huang, Yang-Hsin Shih, Yi-Yen Lee, Henrich Cheng, Ming-Chao Huang

**Affiliations:** a Institute of Pharmacology, School of Medicine, National Yang-Ming University, Taipei, Taiwan; b Neural Regeneration Laboratory, Department of Neurosurgery, Neurological Institute, Taipei Veterans General Hospital, Taipei, Taiwan; c Graduate Institute of Veterinary Pathobiology, College of Veterinary Medicine, National Chung Hsing University, Taichung, Taiwan; d Department of Nursing, Central Taiwan University of Science and Technology, Taichung, Taiwan; e Basic Medical Education Center, Central Taiwan University of Science and Technology, Taichung, Taiwan; f Department of Neurosurgery, Neurological Institute, Taipei Veterans General Hospital, Taipei, Taiwan; g Department of Medicine, National Yang-Ming University, Taipei, Taiwan; h Division of Pediatric Neurosurgery, Neurological Institute, Taipei Veterans General Hospital, Taipei, Taiwan; i Center for Neural Regeneration, Neurological Institute, Taipei Veterans General Hospital, Taipei, Taiwan; j School of Medicine, Taipei Medical University, Taipei, Taiwan

**Keywords:** Leptin, Leptin receptor, Microglia, Root avulsion

## Abstract

Leptin (Lep) is mainly, although not exclusively, secreted by adipocytes. In addition to regulating lipid metabolism, it is also a proinflammatory factor and involved in the development of neuropathic pain after peripheral nerve injuries (PNI) (Lim et al., 2009; Maeda et al., 2009) [1,2]. Leptin or its messenger ribonucleic acid expression has been found in various brain regions normally and in the dorsal horn after PNI (Lim et al., 2009; Ur et al., 2002; La Cava et al., 2004; White et al., 2004) [1,[3], [4], [5]]. However, the expression pattern of Lep and Leptin receptor (LepR) after preganglionic cervical root avulsion (PCRA) is still unknown. We provide data in this article related to Chang et al. (2017) [6]. Here, our data showed a profound Lep and LepR expression in the neurons of dorsal root ganglion (DRG) after PCRA. Moreover, the expression of Lep and LepR were also identified in significant portions of the neurons and microglia located in the dorsal horn. The roles of these increased expressions in the development of neuropathic pain after PCRA deserve further study.

**Specification Table**

**Table:** 

Subject area	Neuroscience
More specific subject area	Leptin (Lep), Leptin receptor (LepR), Neural trauma, spinal cord, dorsal root ganglion (DRG)
Type of data	Image (immunofluorescence)
How data was acquired	Fluorescent microscope (Axioskop 2 Carl Zeiss, LLC, United States)
Data format	Raw
Experimental factors	DRG (Fig. 1) and dorsal horn (DH) of spinal cord (Fig. 2, Fig. 3) was obtained from C57BL/6J mice after preganglionic cervical root avulsion (PCRA)
Experimental features	The DRG and DH were labeled using primary antibodies raised against leptin, its receptor, ionized calcium binding adaptor molecule 1 (Iba1, microglia), neuronal nuclei (NeuN, neuron), and glial fibrillary acidic protein (GFAP, astrocyte).
Data source location	Taipei, Taiwan.
Data accessibility	Data within this article

**Value of the Data**

• Generalized expression of Lep and LepR was noted 7 days after PCRA in DRG neurons. However, it has been reported that around 95% of DRG neurons die after PCRA [7]. The functions and/or effect of the leptin produced by neurons undergoing apoptosis remain to be clarified.
• Substantial intracellular expression of Lep was noted in the neurons of spinal cord after PCRA. It is known that extracellular leptin acts on its receptor located in the cell membrane of neurons. However, the roles of the intraneuronal Lep in the development of neuropathic pain have not been studied.
• Significant amount of Lep was noted in the dorsal horn of the spinal cord in neurons and microglia, but not in astrocytes. The control mechanism and the significance of this *de novo* production of Lep are of interest for future investigation.
• It is reported that LepR has been found to be increased after PNI in neurons, and to a lesser extent in astrocytes [1]. But after PCRA, we found that significant number of microglia has LepR expression. Does this difference be relevant to the different clinical presentation of the neuropathic pain after PNI and PCRA requires further research.

## 1 Data

**Fig. 1:**
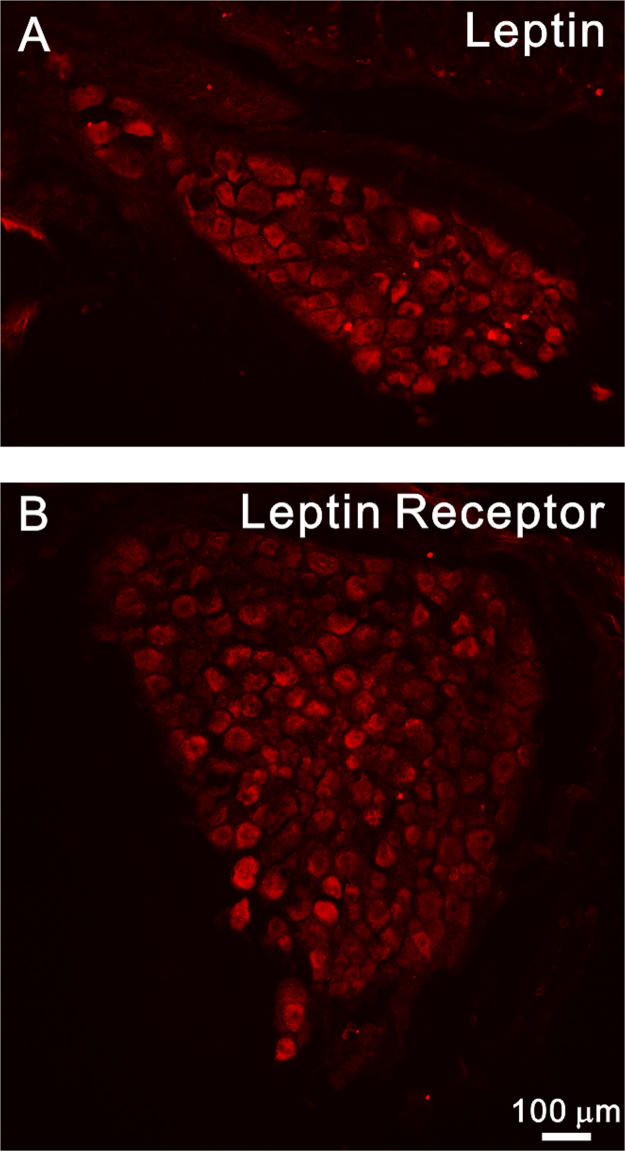
Both leptin (A) and leptin receptor (B) were expressed in the dorsal root ganglion.

**Fig. 2:**
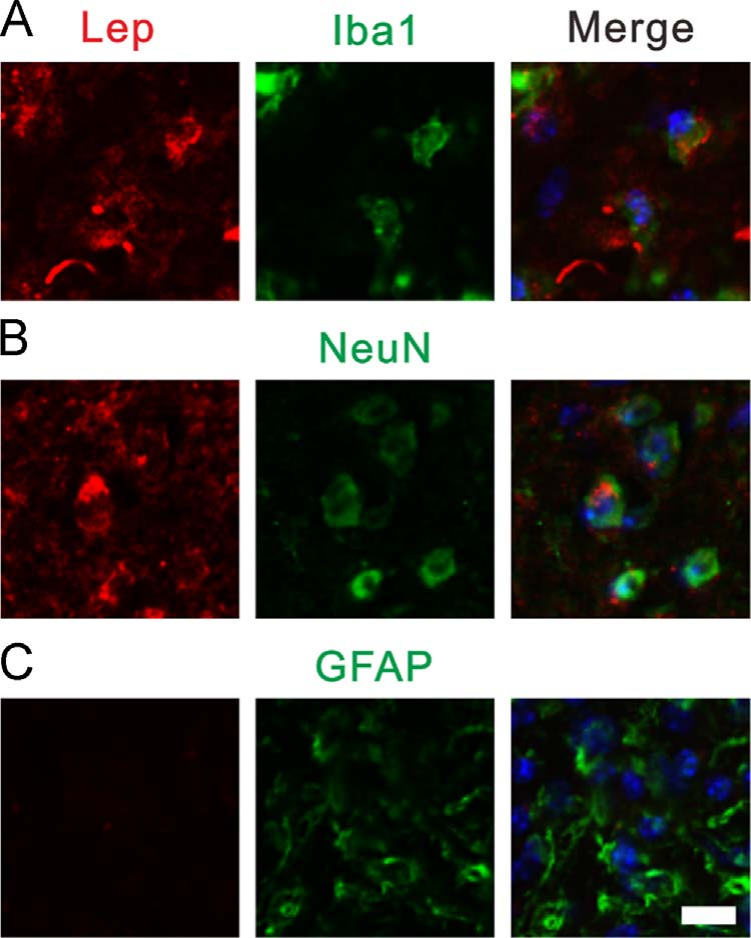
Double immunofluorescence staining of leptin with Iba1, NeuN, and GFAP. (A) Leptin was colocalized with Iba1. (B) Leptin was colocalized with NeuN. (C) Leptin was not colocalized with GFAP. Scale bar, 20 µm.

**Fig. 3:**
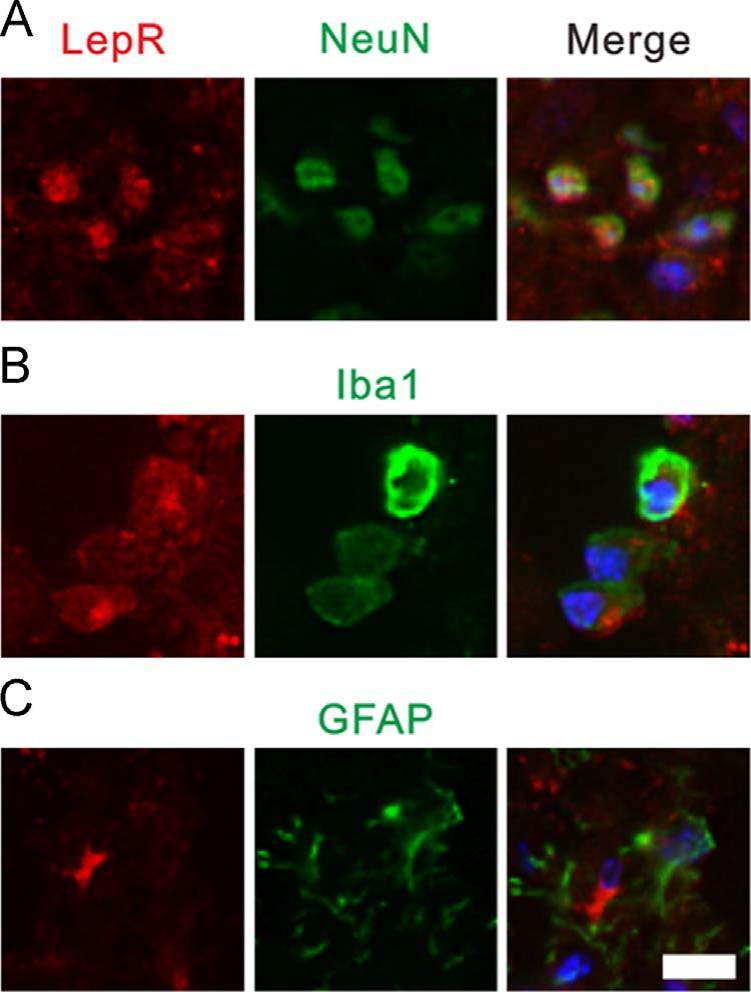
Double immunofluorescence staining of LepR with NeuN, Iba1, and GFAP. (A) NeuN-positive cells were also positive for LepR. (B) Iba1-positive cells were also positive for LepR. (C) GFAP positive cells were not positive for LepR. Scale bar, 20 µm.

We showed that both Lep and LepR were expressed in the DRG on day 7th after PCRA (Fig. 1). By using double immunolabeling for Lep/LepR and markers of specific cell populations, we found that after PCRA, there are increased expression of leptin in the dorsal horn [6]. This increased leptin expression was colocalized with NeuN (19%) and Iba1 (37%), but not with GFAP (Fig. 2). Similarly, increased expression of LepR was also noted [6]. The LepR was also coexpressed with NeuN (56%) and Iba1 (47%), but not with GFAP (Fig. 3).

## 2 Experimental design, materials and methods

### 2.1 Animals

Adult male and female C57BL/6J (*n* = 10) were obtained from the National Laboratory Animal Center in Taiwan. All animals were housed in ventilated and humidity- and temperature-controlled rooms with a 12 h/12 h light/dark cycle. Food and water were provided *ad libitum*. Animals were maintained according to the Guide for the Care and Use of Laboratory Animals of the Institute of Laboratory Animal Resources Commission on Life Sciences (National Research Council, USA). All experimental procedures complied with the Ethical Guidelines of the International Association for the Study of Pain. The protocols were reviewed and approved by the Institutional Animal Care and Use Committee of Taipei Veterans General Hospital, Taiwan.

### 2.2 Surgical procedures

The 13-week-old male and female C57BL/6J mice (23.5 ± 3.8 g) (*n* = 10) were anaesthetized using isoflurane and placed on a heating pad. Rectal temperatures were monitored during surgery. All animals received a left hemi-laminectomy at the cervical 5th–7th vertebrae. After the dura was opened, the left 6th–8th cervical dorsal roots were identified, gently pulled, and avulsed at their junctions with the spinal cord, leaving no proximal stump.

### 2.3 Immunofluorescence

Animals were sacrificed 7 days after surgery. Tissues were post-fixed in 4% paraformaldehyde overnight and kept in 30% sucrose/15% glycerol at 4 °C. Cryosections (10-µm-thick) were stained using standard immunofluorescence methods. Briefly, the sections were blocked in 5% normal donkey serum (Jackson Immuno-Research, West Grove, PA, USA) and then incubated with the following primary antibodies: microglial marker [Iba1 (1:500; #019-19741, Wako Chemicals, Neuss, Germany)], astrocyte marker [GFAP (1:1000; AB5804, chemicon, Merck KGaA, Darmstadt, Germany)], neuronal marker [NeuN (1:100; MAB377, chemicon, Merck KGaA, Darmstadt, Germany)], leptin (1:50; AF498, R&D system, Minneapolis, MN, USA), and the LepR (1:100, AF497, R&D Systems, Minneapolis, MN, USA). The following fluorescent secondary antibodies were then applied: Alexa 488 donkey anti-rabbit, Alexa 488 donkey anti-mouse, Cy3 donkey anti-goat, and Cy3 donkey anti-rabbit (Jackson Immuno-Research, West Grove, PA, USA). The sections were visualized at 200× magnification under a fluorescence (Axioskop 2 Carl Zeiss, LLC, United States) microscope. The numbers of Lep^+^, LepR^+^ with Iba1^+^, GFAP^+^, and NeuN^+^ cells were quantified by counting the percentage of positive cells in the superficial lamina (SL) of the dorsal horn of one or two randomly selected regions in each corresponding area of the spinal cord throughout the 6th–8th levels. In total, 5 sections were selected for each animal. The percentage of Lep and LepR with Iba1, NeuN and GFAP positive cells was counted by dividing the number of Lep, LepR/ Iba1, NeuN and GFAP cells by the number of Iba1, NeuN and GFAP cells. The personnel who were assigned to quantify the results were blinded to the treatment group of the animals in all studies.
