# Supplementary material for: Data on the expression of leptin and leptin receptor in the dorsal root ganglion and spinal cord after preganglionic cervical root avulsion
Source: Data Brief. 2017 Oct 7;15:567–72. doi: 10.1016/j.dib.2017.10.005 (PMC5651484; doi:10.1016/j.dib.2017.10.005)
Supplement: Supplementary file 1 — Transparency document [file mmc1.docx]

**Conflict of interest:**

The authors have no conflicts of interest to declare.
